# Metabonomic Analysis of the Therapeutic Effects of Chinese Medicine Sanqi Oral Solution on Rats With Exhaustive Exercise

**DOI:** 10.3389/fphar.2019.00704

**Published:** 2019-07-04

**Authors:** Peng Xu, Shasha Li, Ruimin Tian, Ling Han, Wei Mao, Li Li, Chuang Li, Yiming Wang, Guoan Luo, Nizhi Yang

**Affiliations:** ^1^Guangdong Provincial Hospital of Chinese Medicine, Guangzhou, China; ^2^Department of Chemistry, Tsinghua University, Beijing, China

**Keywords:** exhaustive exercise, Sanqi oral solution, Radix Astragali, Radix Notoginseng, metabonomic, therapeutic effect, biomarkers

## Abstract

Exhaustive exercise has emerged as an important health issue nowadays. This study was designed to assess the metabolite abnormalities of rats after exhaustive exercise and the holistic efficacy of Chinese medicine Sanqi oral solution (SQ). Through exhaustive swimming, the exhaustive exercise model in rats was established. Thirty male Sprague–Dawley rats were randomly divided into control, model, and treatment groups. SQ (12 mL·kg^−1^·d^−1^) or 0.9% saline solution was administrated orally by gastric gavage. After 4 weeks, serum samples were collected for biochemical measurements and ultra performance liquid chromatography (UPLC)/quadrupole time-of-flight mass spectrometry (Q-TOF-MS)-based metabonomic study. It was found that rats with SQ intervention showed longer exhaustive swimming time (*P* < 0.05) than model rats, with an average of 1,160.36 ± 123.89 s in SQ group and 906.57 ± 172.11 s in model group. Among the biochemical indices, the levels of creatine kinase isoenzyme, lactate dehydrogenase, and glucose of exhaustive exercise rats increased, whereas levels of creatine kinase, urea, triglyceride, and total cholesterol decreased. These biochemical indices came normal after SQ administration, except for triglyceride. Twenty-seven potential biomarkers belonging to sphingolipids, phospholipids, fatty acids, amino acid, and other classes were identified in serum. This study indicated that SQ exerted protective effects on exhaustive exercise by significantly prolonging the swimming endurance time. The metabonomic-based findings of the metabolic state and analysis of potential biomarkers in serum well correlated with biochemical assessment, confirming that SQ had a definite efficacy. Moreover, the shifts in lipid-related metabolites and glycolytic pathway suggested that SQ may serve as a potential supplementation in sports nutrition for its pharmacological effect of regulating energy metabolism as well as improving signal transduction and muscle-cell physiological functions.

## Introduction

Regular and moderate exercise can contribute to the health maintenance, disease prevention, and stress relief (Kingwell, 2000; Reimers et al., 2012). However, exhaustive exercise or athletic overtraining may lead to excessive physical loading, and eventually persistent or relapsing fatigue, or even damages such as oxidative stress (Sen, 1995; Fehrenbach and Northoff, 2001; Popovic et al., 2012; McLeay et al., 2017), local and systemic inflammation (Woods et al., 2006; Baynard et al., 2012; Kohne et al., 2016; Liu et al., 2017), muscle damage (Woods et al., 2006; Camera et al., 2016; Liu et al., 2017), adrenal insufficiency (Brooks and Carter, 2013), or even kidney function failure (Wu et al., 2012; Lin et al., 2013), which can adversely affect the quality of people’s lives. Thus, it is urgently needed to prevent and treat these changes during exhaustive exercise besides scientific diagnosis and assessment.

Many studies have investigated the oxidative changes, muscle damages, or heart histomorphometric analysis after exhaustive exercise, and have tried some nutritional intervention to eliminate exercise-induced fatigue and enhance exercise performance. Traditional Chinese medicine (TCM) has been utilized to effectively treat various diseases for centuries and thought to be the most promising healthcare gift that people present to the whole world. Sanqi oral solution (SQ), consisting of Radix Astragali (*Astragalus membranaceus Fisch. ex Bunge*) and Radix Notoginseng [*Panax notoginseng (Burkill) F.H.Chen*], is a patented formula and ready-made pharmaceutical preparation of Guangdong Provincial Hospital of Chinese Medicine (Cantonese medicine production number: Z20071155) (Wei et al., 2013). In clinical settings, SQ shows protective and therapeutic effect of eliminating fatigue on many chronic diseases including nephrotic syndrome, hypertension, arteriosclerosis, and coronary heart disease. According to the Chinese medicine theory, the onset and process of the aforementioned chronic diseases are closely related to qi deficiency and blood stasis, which are also the typical pathogenesis of exhaustive exercise. SQ has been widely used as prescribed Chinese medicine in clinical practice. However, the holistic efficacy of SQ on exhaustive exercise has not been analyzed.

Metabonomics, which can reflect the global metabolic state of an organism, provides a new perspective on assessing the holistic efficacy and elucidating the therapeutic mechanisms of Chinese medicine (Chen et al., 2007; Ulrich et al., 2007; Lao et al., 2009; Lindon et al., 2010; Everett, 2015; Zhang et al., 2017). As a systematic approach, metabolomics reveals the whole metabolic profile changes of living systems in response to external stimuli such as oxidative stress and drug treatments. It is a combination of data-rich analytical chemical measurements and chemometrics for profiling metabolism in complex systems, typically carried out using biofluids or tissue samples (Lindon et al., 2010; Everett, 2015). Metabonomics involves searching for disease-related potential biomarkers and metabolic pathways by obtaining metabolic fingerprints that contain metabolite information. These fingerprints can be obtained using a series of data collection methods and analysing the metabolites with multivariate statistical techniques (Shulaev, 2006; Xu et al., 2007). This approach has been successfully used to reveal the pathophysiological perturbations of diseases such as cardiovascular disease (Senn et al., 2012), type 2 diabetes (Palau-Rodriguez et al., 2015; Xiang et al., 2015), and renal failure (Won et al., 2016) or to elucidate the possible mechanisms of drug effects (Chen et al., 2007; Lindon et al., 2010; Lu et al., 2011; Everett, 2015; Wishart, 2016).

According to the essence of “treatment based on syndrome differentiation” in Chinese Medicine Theory and our previous clinical practices, SQ may have potential of alleviating fatigue and preventing exhaustion. In order to test the hypothesis, we established the rat model of exhaustive exercise (swimming), detected the time to exhaustion to evaluate the drug efficacy, and used the metabonomics method to investigate the metabolite abnormalities and find potential biomarkers and metabolic pathways related to exhaustive exercise, thus elucidating the underlying mechanisms of SQ for the prevention of exhaustive exercise.

## Materials and Methods

### Preparation and Chemical Analysis of SQ

SQ was provided by Guangdong Provincial Hospital of Chinese Medicine. It has been chemically characterized in Guangdong Provincial Hospital of Chinese Medicine and approved by the Drug Administration of Guangdong Province for production (Cantonese medicine production number: Z20071155). It is extracted from the fixed combinations of two crude drugs: Radix Astragali (0.333 g/ml) and Radix Notoginseng (0.056 g/ml). The botanical, herbal, and Chinese names of ingredients, as well as the instruction of SQ have been listed in **Sup. Figure 1**. Both of their botanical names can be checked and validated by using http://mpns.kew.org/mpns-portal/?_ga=1.111763972.1427522246.1459077346. The quality of herbs and herbal extracts was consistent with the standards of Chinese Pharmacopoeia (China Pharmacopoeia Committee, 2015).

Qualitative and quantitative analysis of SQ was performed on an Agilent 1200 HPLC system with DAD detector. The LC separation was carried out on a Kinetex C18 column (4.6 × 100 mm, 2.6 μm, Phenomenex) at 30°C. The mobile phase consisted of water as solvent A and acetonitrile as solvent B, while the flow rate was 1.8 ml/min. A gradient elution program was applied as follows: 0–12 min, 88%–80% A; 12–26 min, 80%–74% A; 26–40 min, 74%–35% A. The UV detection wavelengths were set at 205 nm and 284 nm.

### Animals and Treatment

Animal experiments were conducted on specific pathogen-free Sprague–Dawley (SD) rats after 3 days of acclimatization. Rats were provided by the Experimental Animal Center of Guangdong Medical (Certification No. 0079361) and housed in Experimental Animal Center of Guangdong Provincial Hospital of Chinese Medicine. All of our experiments were performed in accordance with the internationally accepted standard guidelines for the use of animals and were reviewed and approved by the Institutional Animal Care and Use Committee at Guangdong Provincial Hospital of Chinese Medicine. The rats were housed under standard environmental conditions (23 ± 2°C, 55% ± 5% humidity, and 12 h/12 h light/dark cycle) and allowed free access to water and standard laboratory diet.

In the experiment, 30 male SD rats (180–220 g) were randomly divided into control, model, and treatment groups. All rats were allowed free access to water and food. Rats in the model and treatment groups were forced to swim exhaustively every day and weighed before exhaustive swimming weekly. The rats swam in a 60-cm-deep pool of water at room temperature and were picked up when 50% of the rats were exhaustively submerged in water for 10 s.

After 1 week of adaptive swimming, the treatment group was treated with SQ at a dose of 12 ml·kg^−1^·d^−1^ (the dose has been optimized according to the preliminary experimental results) prior to exhaustive swimming, whereas the control and model groups were both treated with 0.9% saline solution for 28 days. The exhaustive swimming time of the model and treatment groups was measured during the drug intervention period.

### Sample Preparation

Blood samples were collected from the abdominal aorta after 1 h of final administration and then centrifuged at 3,000 rpm for 10 min at room temperature. The supernatant obtained was divided into two parts: one for serum biochemical detection and the other for serum metabonomic analysis. Sera were immediately frozen, stored at −80°C, and thawed before analysis.

Before the metabonomic analysis, 1,200 μL of methanol was added to 400 μL aliquots of serum. The mixture was vortex mixed for 2 min and centrifuged at 12,000 rpm for 15 min at 4°C. The clear supernatant was transferred and evaporated to dryness by N_2_. The dried sample was diluted with 300 µL of methanol-H_2_O (80:20, v/v) and filtered through a 0.22-µm membrane filter for UPLC/Q-TOF-MS analysis. Approximately 5 μL of the sample was injected into the column in each run.

### Reagents and Materials

The acetonitrile used for LC and MS analyses was LC grade from Fisher (USA). The formic acid for MS analysis was LC grade from Acros (USA).The water for LC and MS analyses was purified using a Milli-Q academic water purification system (Millipore, Milford, MA, USA). SQ was provided by the Department of Nephrology of Guangdong Provincial Hospital of Chinese Medicine (product lot 071101).

### UPLC Conditions

The separations were carried out on a Waters Acquity UPLC BEH C_18_ column (2.1 mm × 100 mm, 1.7 μm) at 30°C. The mobile phase consisted of acetonitrile as solvent A and 0.1% formic acid in water as solvent B, while the flow rate was 0.4 mL/min. The gradient programs used for separation were as follows: 0–5 min, 2%–50% A; 5–7 min, 50%–60% A; 7–14 min, 60%–65% A; 14–18 min, 65%–80% A; 18–24 min, 80%–90% A; 24–26 min, 90%–95% A; 26–28 min, 95%–2% A; 28–30 min, 2% A. The injection volume of the test sample was 5 µL. Each sample was injected three times. Each wash cycle consisted of 200 μL of strong wash solvent (80% CH_3_CN-H_2_O, 8:2, v/v) and 600 μL of weak wash solvent (10% CH_3_CN-H_2_O, 1:9, v/v).

### MS Conditions

MS was carried out on a Waters Q-TOF Premier with an electrospray ionization system (Waters MS technologies, Manchester, UK). Electrospray ionization-mass spectrometry (ESI-MS) spectra were acquired in both positive- and negative-ion V modes. The ESI-MS analysis conditions were as follows. The capillary voltages were set to 3.0 and 2.5 kV for the positive- and negative-ion modes, and the sample cone voltages were 30 and 45 V for the positive- and negative-ion modes, respectively. The desolvation gas flow was set to 600 L·h^−1^ at a desolvation temperature of 350°C, the cone gas was set to 50 L·h^−1^, and the source temperature was 110°C. The Q-TOF Premier acquisition rate was 0.1 s with a 0.02-s inter-scan delay. The instrument was operated with the first resolving quadrupole in a wide-pass mode (50–1,500 Da). Argon was used as the collision gas.

All analyses were performed using a lock spray to ensure accuracy and reproducibility. Leucine-enkephalinamide acetate was used as the lock mass ([M-H]^−^ = 553.2775, [M+H]^+^ = 555.2931) at a concentration of 200 рg·μL^−1^ and flow rate of 20 μL·min^−1^.

### Data Analysis

Data preprocessing was performed with MassLynx applications manager version 4.1 (Waters, MA, USA). The processed data list was exported and processed by principle component analysis (PCA) and partial least-squares discriminant analysis (PLS-DA) using the software package SIMCA-P version 12.0 (Umetrics AB, Umeå, Sweden).

Statistical analyses were performed using the SPSS software (version 11.5, USA). Assumptions of normality and homogeneity of variance were first verified. Data were presented as the mean ± standard deviation for continuous variables with a normal distribution. The independent sample *t*-test or one-way ANOVA was used to analyze the differences among groups for continuous measures. *P* values < 0.05 were considered statistically significant. All probability values were two sided.

## Results and Discussion

### Quality Control of SQ

The high performance liquid chromatography (HPLC) analysis identified the major components in SQ, as indicated in **Sup. Figure 2** and **Sup. Table 1**.

### Effects on Weight and Exhaustive Swimming Time of Rats

**Figure 1:**
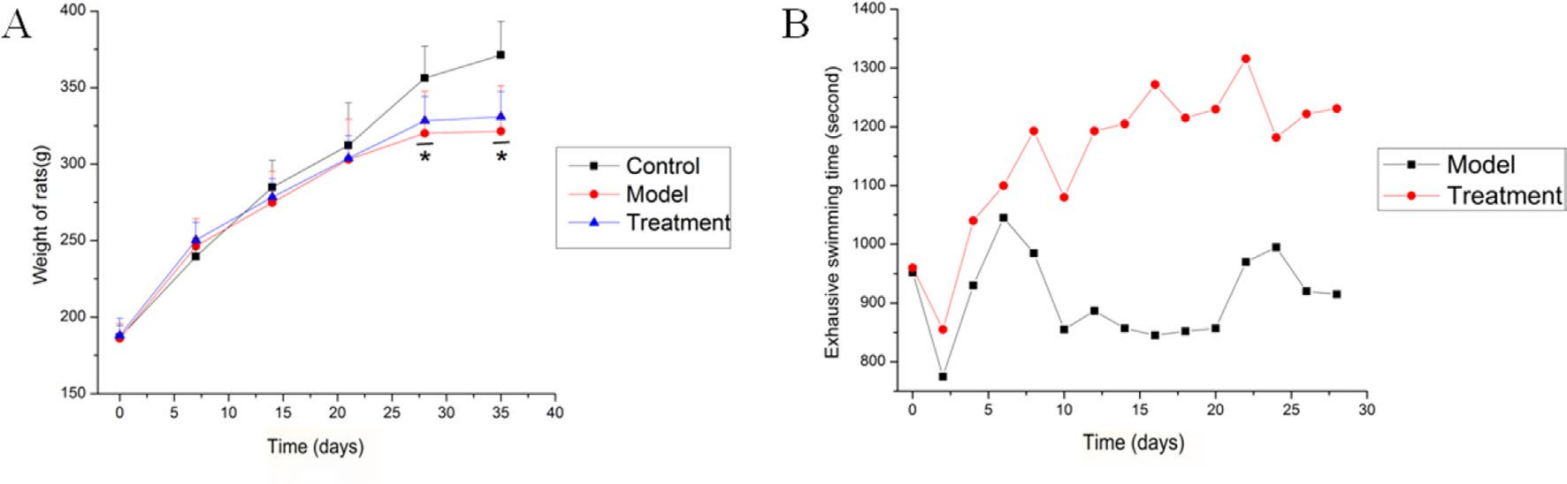
Weight and exhaustive swimming time of rats from the start to the end of the experiment. **(A)** Weight of rats were measured weekly. Data were described as the mean ± standard deviation for 10 rats in each group. *P < 0.05, compared with the control group. **(B)** Exhaustive swimming time of the model and treatment groups was measured during the drug intervention period. Data were reported as count for each group. The time was calculated from the point when rats were placed in water to the point when 50% of the rats were exhaustively submerged in water for 10 s.

Ten rats for each group were included in this study. The weight of all rats was recorded every week, and the exhaustive swimming time of rats in both model and treatment groups was measured every 2 days. **Figure 1A** shows that the weight of all rats increased with time. Starting from the second week, the weight growth rate of the control group was faster than those of the other groups, and this phenomenon persisted until the end of the experiment. The Dunnett’s test showed that the weights of the rats in each group were equal at the beginning of the experiment. However, the final weights of the model and treatment groups were significantly lower than that of the control group (*P* = 0.0001 and 0.001, respectively). No significant difference was observed between the model and treatment groups. **Figure 1B** shows that the exhaustive swimming time of the treatment group was equal to that of the model group during the adaptive swimming period. After drug intervention (0.9% saline solution for the model group and SQ for the treatment group) for 6 days, the average swimming time was 1,200.0 ± 42.4 s for the treatment group, while 945.0 ± 63.6 s for the model group. From the sixth day to the end of the experiment, the treatment group always had a longer swimming time than the model group (**Figure 1B**, 906.57 ± 172.11 s in model group and 1,160.36 ± 123.89 s in SQ group), indicating that SQ promoted physical recovery and increased the anti-fatigue capacity of the rats.

### Serum Biochemistry

The concentrations of serum biochemical indices were also determined, and the results are listed in **Table 1**. Compared with the control group, the levels of creatine kinase isoenzyme (CK-MB), lactate dehydrogenase (LDH), and glucose (GLU) of the model group increased, whereas their levels of creatine kinase (CK), urea (UREA), triglyceride (TG), and total cholesterol (CH) decreased. No statistical differences in CK, GLU, and TG (**Table 1**) were observed by the independent sample *t*-test. After treatment, the levels of biochemical indices of the treatment group, except for triglyceride, were equal to that of the control group; however, no statistical difference was observed between the two groups. This result revealed the recovery of the treatment group and suggested the regulative effect of SQ on rats with exhaustive exercise.

**Table 1:** Comparison of serum biochemistry among groups.*^a^*

	Control group	Model group*^b^*	Treatment group*^b^*
**No. of subjects**	10	10	10
**CK (U L^−1^)**	431.71 ± 74.94	461.80 ± 52.35	486.86 ± 47.01
**CK-MB (U L^−1^) **	1,192.90 ± 114.36	1,321.75 ± 123.96*	1,172.22 ± 130.21^Δ^
**LDH (U L^−1^)**	1,220.89 ± 69.88	1,288.50 ± 57.61*	1,199.50 ± 99.76^Δ^
**UREA (mmol L^−1^)**	2.83 ± 0.49	2.12 ± 0.51**	2.93 ± 0.52^ΔΔ^
**GLU (mmol L^−1^)**	3.77± 1.70	4.70 ± 1.37	3.60 ± 1.51
**TG (mmol L^−1^)**	0.69 ± 0.15	0.41 ± 0.03***	0.39 ± 0.07***
**CH (mmol L^−1^)**	1.26 ± 0.18	1.21 ± 0.12	1.36 ± 0.11^ΔΔ^

^a^ The serum levels of biochemical indices of rats are presented as the mean ± standard.

^b^ The serum concentrations of biochemistry were statistically tested by Dunnett’s t-test when the variance for different groups was homogeneous and by the Games–Howell test when the variance is nonhomogeneous. *P < 0.05, **P < 0.01, ***P < 0.001 compared with the control group; ^Δ^P < 0.05, ^ΔΔ^P < 0.01 compared with the model group.

The concentrations of CK, CK-MB, and LDH are closely related to cell integrity. These substances are macromolecular proteins that are mainly present in tissue cells and transferred into the circulatory system when muscle cells are damaged. CH, which is involved in maintaining membranes, intracellular transport, and nerve conduction, is also a precursor molecule in several biochemical pathways. The increase in CK, CK-MB, and LDH, as well as the decrease in CH of the model group implied that muscle cell injuries were induced by excessive exercise. However, the serum levels of CK and CH did not significantly differ from the control group. After SQ intervention, the levels of these four indices were equal to those of the control group, indicating the restoration of damaged cells.

The GLU level of the model group increased, reflecting stress-induced increase in energy metabolism under long-term excessive movement. Physical fatigue is acknowledged to be due to the lack of energy. The body of fatigued rats cannot produce sufficient energy, leading to a metabolic dysfunction, which possibly reduced the level of urea of the model group because urea is the final product of protein metabolism. The normal GLU and urea levels of the treatment group revealed the effect of SQ on regulating the metabolism of rats with exhaustive exercise.

Unexpectedly, the TG level of the treatment group did not become normal. This result may be due to the consumption of TG to gain energy for the considerable requirements of daily physical activities. Consequently, the TG concentrations of the model and treatment groups were similar, and both were lower than that of the control group (**Table 1**).

### Metabonomic Study

#### Establishment of Metabolic Fingerprints

**Figure 2:**
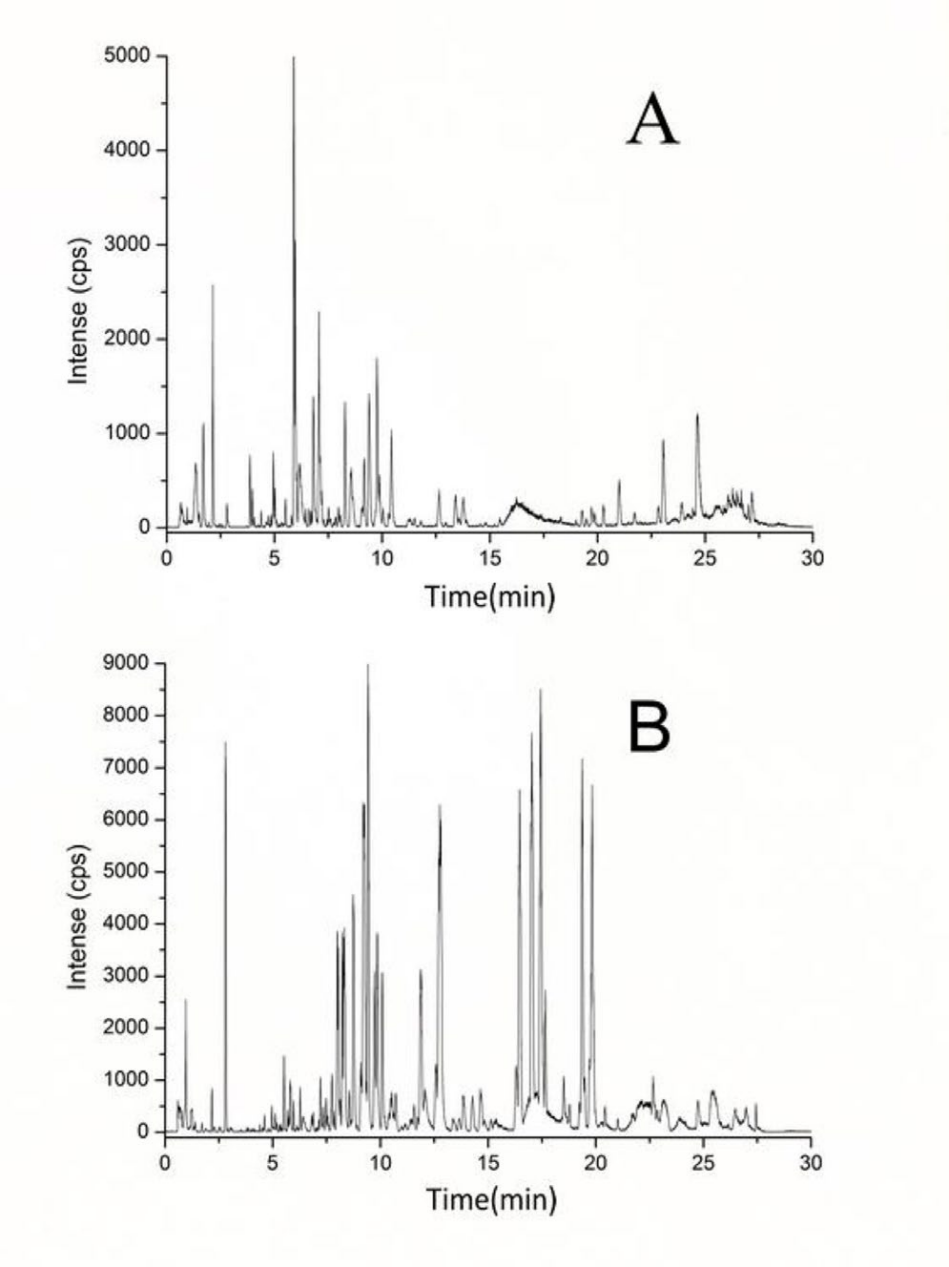
Representative base peak intensity chromatogram of rat serum obtained in the electrospray ionization (ESI) positive-ion **(A)** and negative-ion **(B)** V modes based on ultra performance liquid chromatography/quadrupole time-of-flight mass spectrometry (UPLC/Q-TOF-MS).

Serum metabolic profiling was performed to explore the potential biomarkers and metabolic pathways related to exhaustive exercise. Adequate information may lay an important foundation to guarantee the detection of metabolites. Full-scan detection was set in both ESI positive and negative modes to acquire the maximum amount of detectable metabolites. Based on the optimal UPLC and MS conditions (described in MATERIALS AND METHODS), the UPLC-MS base peak intensity chromatograms of serum were obtained in the ESI positive- and negative-ion V modes (**Figures 2A** and **2B**, respectively). **Figure 2** shows that abundant chromatographic peak information was obtained in each ion mode and that the positive-ion mode was more sensitive for identifying substances within the interval of 3–11 min, whereas the negative-ion mode was suitable within 5–20 min.

The injection precision reflected the stability of the analysis, which was important to guarantee the reliability of the acquired data. The variability evaluation was carried out by the continuous analysis of six injections of the same samples. The relative standard deviations (RSDs) of the relative retention time and relative peak area were below 3.0%. In order to determine the influence of sample preparation on the stability of data, six parallel samples were prepared using the same preparation protocol. The RSD of the relative retention time and peak intensity for the main peaks were less than 4.5%. The test precision of sample stability was determined with one sample during 24 h, during which the solution was stored at 4°C. The RSDs of the relative retention time and relative peak area were below 3.8%. The resulting data showed that the precision and repeatability of the proposed method were satisfactory for metabonomic analysis.

#### PCA and PLS-DA Processing of UPLC-MS Data

**Figure 3:**
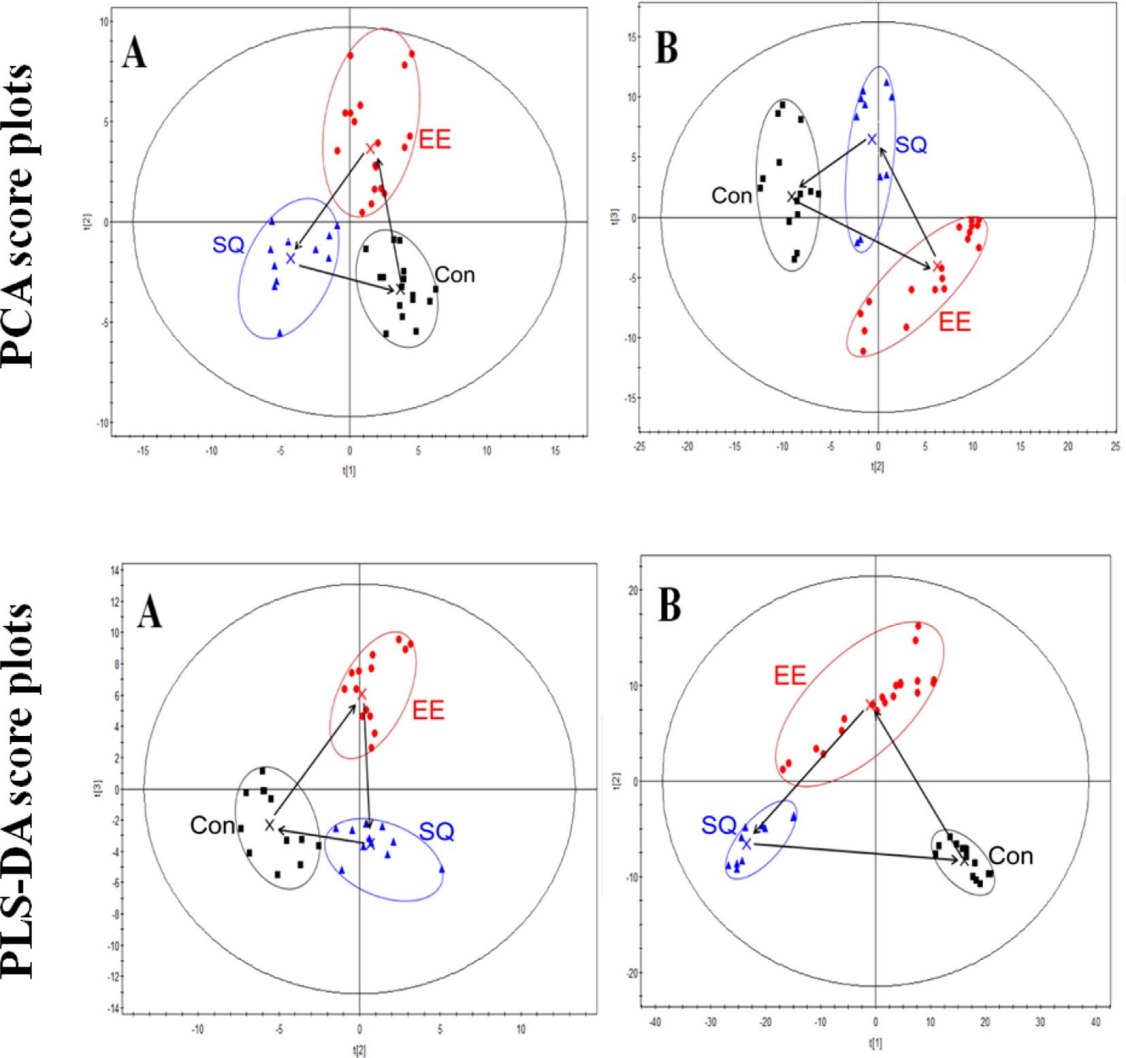
Principle component analysis (PCA) and partial least-squares discriminant analysis (PLS-DA) score plots of the serum data of the control, model, and treatment groups in the ESI positive-ion **(A)** and negative-ion **(B)** V modes based on UPLC/TOF-MS. Con, control group; EE, exhaustive exercise (swimming) smodel group; SQ, treatment group.

Based on the fingerprints in both positive- and negative-ion modes, PCA and PLS-DA were carried out to distinguish between classes, and the results exhibited satisfactory classification (**Figure 3**). PCA was used first to determine the general interrelation between groups. PLS-DA was subsequently used to maximize the differences in metabolic profiling. After processing by PCA and PLS-DA, the mean-centered PCA and PLS-DA score plots were generated to trace and compare the variations among the metabolic events in rats.

In the PCA and PLS-DA maps, each spot represented a sample and each assembly of samples indicated a particular metabolic pattern of the different groups. The locus marked by arrows represented the trend of the mean metabolite pattern change. **Figure 3** showed that the metabolic state of the model group was far from the initial position of the control group. This finding indicated that exhaustive swimming exercise disturbed endogenous substance metabolism and significantly altered the metabolic fingerprints of serum compared with the normal state. After treatment, the metabolic state of the treatment group gradually moved to the space of the control group, indicating the recovery of the disturbed metabolism state. The metabolic state of the treatment group moved to the state of control group in the PCA or PLS-DA scores plot, but the relative biomarker content of the treatment group did not return to the normal level, indicating the negative effect of excessive exercise on the rats.

#### Identification of Potential Biomarkers

According to a threshold of variable importance generated after PCA and PLS-DA processing, combined with the *t*-test, significantly different metabolites were selected (the corresponding variable importance in the projection (VIP) > 1.5) for further study. The possible elemental compositions of the selected compounds were generated using the software MassLynx according to the calculated mass, mass deviation (mDa and ppm), double bond equivalent, formula, and isotopic pattern of the selected ion (*i*-fit value). A lower *i*-fit value and a smaller mass deviation indicate a more accurate elemental composition.

The structural information of the selected metabolites was obtained by searching the freely accessible databases HMDB (http://www.hmdb.ca) and KEGG (http://www.genome.jp). Consequently, 27 potential biomarkers belonging to sphingolipids, phospholipids, fatty acids, amino acids, and other classes were identified and are listed in **Table 2**. The fatty acids accounted for a relatively large proportion, comprising 11 substances: eicosapentaenoic acid (EPA), 8, 9-epoxyeicosatrienoic acid (8, 9-EET), 15(S)-hydroxyeicosatrienoic acid (15(S)-HETrE), palmitoleic acid, docosahexaenoic acid (DHA), arachidonic acid (AA), linoleic acid, docosapentaenoic acid (DPA), palmitic acid, oleic acid, and stearic acid. Four sphingolipids [Cer (d18: 0/24: 0), phytosphingosine, sphinganine, and sphingosine 1-phosphate] and four LysoPCs {LysoPC [18:2 (9Z, 12Z)], LysoPC (16:0), LysoPC (18:0), and LysoPC (P-18:0)} comprised the main portion of the remaining potential biomarkers.

**Table 2:** Identification of significantly different endogenous metabolites in sera of the control, ES, and SQ groups*^a^*.

No.	*t*_R (min)_	*m*/*z*	Ion	Formula	Identification results*^a^*	Model*^b^*	Treatment*^b^*	Treatment*^c^*
1	2.1368	205.0941	[M+H]^+^	C_11_H_12_N_2_O_2_	d-Tryptophan	+(↑)	+(↑)	—
2	3.8640	218.2063	[M+H]^+^	C_10_H_23_N_3_O_2_	Deoxyhypusine	+(↑)	+(↑)	+(↑)
3	5.9727	318.2992	[M+H]^+^	C_18_H_39_NO_3_	Phytosphingosine*^d^*	—	—	—
4	6.6255	330.2608	[M+H]^+^	C_18_H_35_NO_4_	4,8-Dimethyl nonanoylcarnitine	+(↓)	+(↑)	+(↑)
5	7.0912	302.3039	[M+H]^+^	C_18_H_39_NO_2_	Sphinganine	+(↑)	—	—
6	7.1987	380.2525	[M+H]^+^	C_18_H_38_NO_5_P	Sphingosine 1-phosphate	+(↑)	+(↑)	+(↑)
7	8.2913	520.339	[M+H]^+^	C_26_H_50_NO_7_P	LysoPC(18:2(9Z,12Z))	—	+(↓)	+(↓)
8	9.1939	496.3387	[M+H]^+^	C_24_H_50_NO_7_P	LysoPC(16:0)	+(↓)	+(↓)	+(↓)
9	9.4067	303.2271	[M+H]^+^	C_20_H_30_O_2_	Eicosapentaenoic acid	—	—	+(↑)
10	9.8883	480.3397	[M+H]^+^	C_24_H_50_NO_6_P	LysoPC(P-16:0)	+(↑)	+(↑)	+(↑)
11	9.9015	149.0209	[M+H]^+^	C_5_H_8_O_3_S	2-Oxo-4-methylthiobutanoic acid	—	+(↑)	+(↑)
12	10.4449	508.3712	[M+H]^+^	C_26_H_54_NO_6_P	LysoPC(P-18:0)	+(↑)	+(↑)	+(↑)
13	21.0261	319.2581	[M+H]^+^	C_21_H_34_O_2_	Allopregnanolone	—	+(↑)	+(↑)
14	2.8004	650.9106	[M-H]^—^	C_42_H_85_NO_3_	Cer(d18:0/24:0)	+(↑)	+(↑)	+(↑)
15	2.7966	322.9522	[M-H]^—^	C_6_H_14_O_11_P_2_	Fructose 1,6-bisphosphate	+(↑)	+(↑)	+(↑)
16	5.828	407.2832	[M-H]^—^	C_24_H_40_O_5_	Cholic acid	+(↓)	+(↑)	+(↑)
17	7.9959	504.3172	[M-H]^—^	C_25_H_48_NO_7_P	LysoPE(20:2(11Z,14Z)/0:0)	+(↑)	+(↓)	+(↓)
18	9.428	319.2297	[M-H]^—^	C_20_H_32_O_3_	8,9-Epoxyeicosatrienoic acid	—	+(↑)	+(↑)
19	10.0905	321.2483	[M-H]^—^	C_20_H_34_O_3_	15(S)-Hydroxyeicosatrienoic acid	+(↓)	+(↓)	—
20	16.2902	253.2177	[M-H]^—^	C_16_H_30_O_2_	Palmitoleic acid	—	+(↑)	+(↑)
21	16.4444	327.2363	[M-H]^—^	C_22_H_32_O_2_	Docosahexaenoic acid	—	+(↑)	+(↑)
22	17.0048	303.2369	[M-H]^—^	C_20_H_32_O_2_	Arachidonic acid	+(↑)	+(↑)	+(↑)
23	17.4128	279.2365	[M-H]^—^	C_18_H_32_O_2_	Linoleic acid	+(↑)	+(↑)	+(↑)
24	17.6398	329.2503	[M-H]^—^	C_22_H_34_O_2_	Docosapentaenoic acid	+(↓)	—	+(↑)
25	19.3773	255.2356	[M-H]^—^	C_16_H_32_O_2_	Palmitic acid	—	+(↑)	+(↑)
26	19.8058	281.251	[M-H]^—^	C_18_H_34_O_2_	Oleic acid	—	+(↑)	+(↑)
27	22.6847	283.2641	[M-H]^—^	C_18_H_36_O_2_	Stearic acid	+(↑)	+(↑)	+(↑)

^a^ Metabolites were confirmed by t_R_ and m/z with authentic chemicals.

^b^ Compared with the control group.

^c^ Compared with the model group.

^d^ No significant differences (P < 0.05) between any two groups, but P < 0.1 compared among groups.

“↑” indicates a higher level of metabolites, whereas “↓” represents a lower level of metabolites. All data represented the intensity values of metabolites. Data were statistically tested by Dunnett’s test when variance was homogeneous and by Games–Howell test when variance was nonhomogeneous. “+” indicates a statistically significant difference (P < 0.05), whereas “—” represents no statistically significant difference.

#### Functional Pathway and Relationship with Exhaustive Exercise

**Figure 4:**
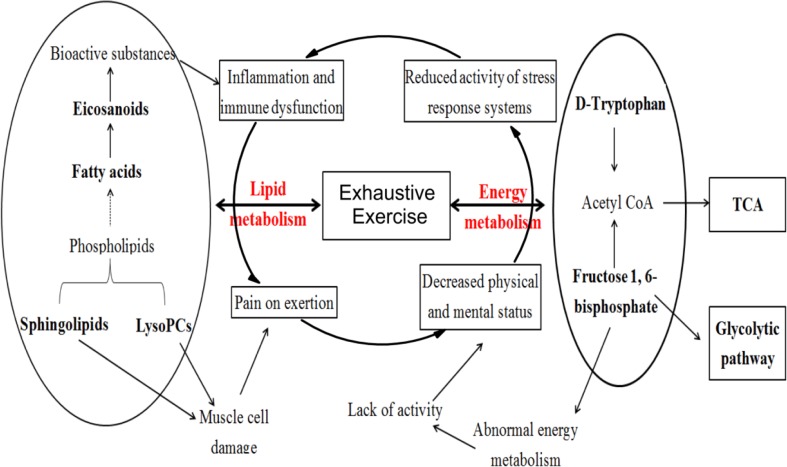
Potential biomarkers and metabolic pathways related to exhaustive exercise.

**Figure 4** shows the pathological status with exhaustive exercise exhibited symptoms of decreased physical status, muscular soreness, neuropsychiatric symptoms, immunological abnormalities, as well as other nonspecific symptoms directly or indirectly related to abnormal energy metabolism, muscle cell damage, inflammation, and dysfunction of immune system from the pathophysiological aspect.

Previous studies have demonstrated that exhaustive exercise can induce free radicals *in vivo* and lead to oxidative stress and inflammatory responses (Woods et al., 2006; Liu et al., 2017; McLeay et al., 2017). Our study showed that the potential biomarkers were mainly substances involved in lipid metabolism, including sphingolipids, phospholipids, and fatty acids. Apart from palmitic acid and stearic acid, the other fatty acids were all unsaturated and increased most obviously for the treatment group. Among these fatty acids, EPA, 8, 9-EET, 15S-HETrE, and AA are eicosanoids, which can exert complex control over many bodily systems, mainly over inflammation or immunity, and act as messengers of the central nervous system (Suzuki et al., 2002; Umamaheswaran et al., 2018). EPA, DHA, and DPA are omega-3 long-chain polyunsaturated fatty acids that are important in maintaining the normal physiological functions of cells and improving blood microcirculation (Ruxton et al., 2004; Riediger et al., 2009). Sphingolipid and LysoPC are also necessary in the maintenance of cell structure and function. Sphingolipids regulate cell growth, differentiation, death, and many important signal transduction pathways (Bartke and Hannun, 2009). LysoPCs may promote oxidative stress (Yasunari et al., 2001; Bouzid et al., 2014), precurse tissue fibrosis (Rindlisbacher et al., 2018), and enable cell structural and functional abnormalities that ultimately induce hemodynamic disorders by inhibiting the activity of Na/K-ATPase (Oishi et al., 1990). A significant increase in fatty acids and sphingolipids, as well as a decrease in LysoPCs after SQ administration indicated the protective effectiveness of SQ on EE from the aspect of increasing energy-related substances and regulating signal transduction and physiological functions of muscle cells. As for the energy metabolism, fructose-1,6-bisphosphate, which is involved in glycolysis metabolism, has been reported to be effective in preventing some damage during ischemia and overoxidation (Juergens and Hardin, 1996; Peeters et al., 2017). The enhanced level of fructose-1,6-bisphosphate confirmed the effect of SQ on protecting the oxidative damage of rats with exhaustive exercise.

## Conclusion

The prolonged swimming endurance time and metabonomic-based findings such as PCA and PLS-DA plots of metabolic states combined with the analysis of potential serum biomarkers suggested that SQ had a protective effect on exhaustive exercise. These results were confirmed by basic characteristics such as exhaustive swimming time and serum biochemistry. The functional study of potential biomarkers and related metabolic pathways revealed that SQ regulated the energy metabolism, improved the signal transduction, and maintained the normal physiological functions of rats with exhaustive exercise. Therefore, we believe that such a metabonomic-based approach may dissect the holistic efficacies of Chinese medicine in treating exhaustive exercise and provide a new perspective on exploring the potential mechanisms of traditional formulas. Further studies need to verify the metabolite differences and perform corresponding mechanism studies for better understanding and management of exhaustive exercise.

## Ethics Statement

In this study, all of the experimental procedures involving animals and their care were performed in accordance with the internationally accepted standard guidelines for the use of animals, and were reviewed and approved by the Institutional Animal Care and Use Committee at Guangdong Provincial Hospital of Chinese Medicine.

## Author Contributions

NY and GL conceived and designed the experiments; PX, SL, and LL performed the experiments; RT, LH, WM, and CL analyzed the data; PX, SL, and RT wrote the manuscript; YW, GL, and NY provided valuable suggestions on the first edition of the manuscript and helped to revise the manuscript. All authors have read and approved the manuscript for publication.

## Funding

This research was funded by National Natural Science Foundation of China (Nos. 81774216 and 81072784), Administration of Traditional Chinese Medicine of Guangdong Province (No. 20181133), and Science and Technology Planning Project of Guangdong Province (Nos. 2016A020226042 and 2018B030322012).

## Conflict of Interest Statement

The authors declare that the research was conducted in the absence of any commercial or financial relationships that could be construed as a potential conflict of interest.

## Abbreviations

TCM, traditional Chinese medicine; SQ, Sanqi oral solution; CK-MB, creatine kinase isoenzyme; LDH, lactate dehydrogenase; GLU, glucose; CK, creatine kinase; TG, triglyceride; CH, total cholesterol; EPA, eicosapentaenoic acid; 8, 9-EET,8, 9-epoxyeicosatrienoic acid; 15(S)-HETrE, 15(S)-hydroxyeicosatrienoic acid; DHA, docosahexaenoic acid; AA, arachidonic acid; DPA, docosapentaenoic acid.
