# Supplementary material for: Metabonomic Analysis of the Therapeutic Effects of Chinese Medicine Sanqi Oral Solution on Rats With Exhaustive Exercise
Source: Front Pharmacol. 2019 Jul 4;10:704. doi: 10.3389/fphar.2019.00704 (PMC6620568; doi:10.3389/fphar.2019.00704)
Supplement: Supplementary file 1 [file Table_1.docx]

**Supplementary materials**


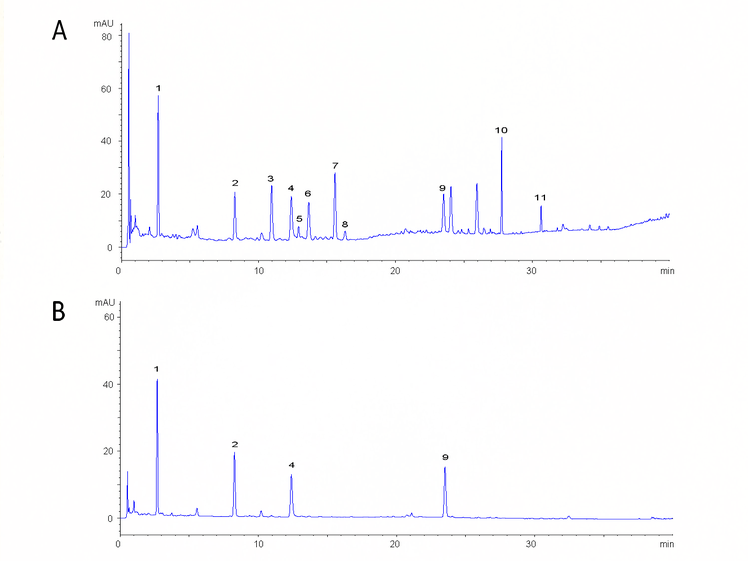


**Fig. 1. UV chromatogram of SQ at 205 nm (A) and 284 nm (B)**

11 peaks were identified as: (1) caylcosin-7-O-β-D-glucopyranoside; (2) ononin; (3) 6aR, 11aR)-3-hydroxy-9,10-dimethoxypterocarpan-3-O-β-D- glucopyranoside; (4) calycosin; (5) notginsenoside R1; (6) isomucronulatol-7-O-β-D-glucopyranoside; (7) ginsenoside Rg1; (8) ginsenoside Re; (9) formononetin; (10) ginsenoside Rb1; (11) ginsenoside Rd. Among these, six compositions were analyzed quantitatively and the result was listed in table 1.

**Table 1.** The content of compounds in 10 mL SQ

| No. | Compound | Content | Detection wavelengths |
| --- | --- | --- | --- |
| 1  2  4  5  7  10 | caylcosin-7-O-*β*-D-glucopyranoside  ononin  calycosin  notginsenoside R_1_  ginsenoside Rg_1_  ginsenoside Rb_1_ | 0.377 mg  0.072 mg  0.255 mg  2.716 mg  7.924 mg  6.160 mg | 284 nm  284 nm  284 nm  205 nm  205 nm  205 nm |
